# EEG/MEG Signal Processing

**DOI:** 10.1155/2007/97026

**Published:** 2008-02-12

**Authors:** A. Cichocki, S. Sanei

**Affiliations:** ^1^Laboratory for Advanced Brain Signal Processing, RIKEN Brain Science Institute, Saitama 351-0198, Japan; ^2^Centre of Digital Signal Processing, Cardiff University, Cardiff CF24 3AA, Wales, UK

Since its invention by the Hans Berger of the electroencephalography (EEG) in 1929, it was strong scientific curiosity in analysis of human brain activity. In fact, the electroencephalography (EEG) and magnetoencephalography (MEG) have developed into one of the most important and widely used quantitative diagnostic tools in analysis of brain signals and patterns. EEG and MEG potentially contain a rich source of information related to functional, physiological, and pathological status of the brain. In particularly, they are essential for the identification of mental disorders and brain rhythms extremely useful for the diagnosis and monitoring of brain activity and offer not only the functional but also pathological, physiological, and metabolic changes within the brain and perhaps other parts in the body.

Recording and analysis of the EEG and MEG now involve a considerable amount of signal processing; for S/N enhancement, feature detection, source localization, automated classification, compression, hidden information extraction, and dynamic modeling. These involve a variety of innovative signal processing methods, including adaptive techniques, time-frequency and time-scale procedures, artificial neural networks and fuzzy logic, higher-order statistics and nonlinear schemes, fractals, hierarchical trees, Bayesian approaches, and parametric modeling. This special issue contributes to the current status of EEG and MEG signal processing and analysis, with particular regard to recent innovations. It reports some promising achievements by academic and commercial research institutions and individuals, and provides an insight into future developments within this exciting and challenging area of functional brain imaging.

Noninvasive functional brain imaging has become an important tool used by neurophysiologists, cognitive psychologists, cognitive scientists, and other researchers interested in brain function. In the last five decades the technology of noninvasive functional imaging has flowered, and researchers today can choose from EEG, MEG, PET, SPECT, MRI, NIRS and fMRI. Each method has its own strengths and weaknesses. Development of signal processing tools mitigates the problems and alleviates some of the weaknesses.

This issue includes the following contributions which cover a wide range of signal processing techniques for analysis, understanding, and recognition of EEG/MEG information.

The first paper, “Canonical Source Reconstruction for MEG” by J. Mattout et al., describes a new, simple but efficient solution to the problem of reconstructing electromagnetic sources into a canonical or standard anatomical space. Electromagnetic lead fields are computed using the warped mesh; in conjunction with a spherical head-model (which does not rely on individual anatomy). The ensuing forward model is inverted using an empirical Bayesian scheme that was described previously in several publications. This enables the pooling of data from multiple subjects and the reporting of results in stereotactic coordinates. Furthermore, it allows the graceful fusion of fMRI and MEG data within the same anatomical framework.

The second paper, “A subspace method for dynamical estimation of Evoked Potentials” by S. Georgiadis et al., describes method for single-channel trial-to-trial EP characteristics estimation. Prior information about phase-locked properties of the EPs is assessed by means of estimated signal subspace and eigenvalue decomposition. Then for those situations that dynamic fluctuations from stimulus-to-stimulus could be expected, prior information can be exploited by means of state-space modeling and recursive Bayesian mean square estimation methods (Kalman filtering and smoothing). The authors demonstrate that a few dominant eigenvectors of the data correlation matrix are able to model trend-like changes of some component of the EPs, and that Kalman smoother algorithm is to be preferred in terms of better tracking capabilities and mean square error reduction. They also demonstrate the effect of strong artifacts, particularly eye blinks, on the quality of the signal subspace and EP estimates by means of Independent Component Analysis (ICA) applied as a prepossessing step to the multichannel measurements.

The third paper, “Inferring functional brain states using temporal evolution of regularized classifiers,” by A. Zhdanov et al., proposes a framework for functional brain state inference problem that utilizes the temporal information present in the brain signals. This application suggests that the relation between the regularization parameters and the temporal profile of the classifier helps improving the classifier accuracy.

In the fourth paper, “Removing ocular movement artefacts by a Joint Smoothened Subspace Estimator (JSSE),” by R. Robert Phlypo et al., a joint smoothened subspace estimator calculates the low and high order statistic information subject to the constraint that the resulting estimated ocular movement artifact source is smooth in time domain. This results in combination of blind source separation with different order statistics. The results have been compared to those of well known blind source separation methods and have shown the capability of the system in mitigating the ocular artefacts automatically.

The fifth contribution, “A framework to support automated classification and labeling of brain electromagnetic patterns,” by G. A. Frishkoff et al., focuses on patterns in averaged EEG (ERP) data to define high-level rules and concepts for ERP components and to design an automated data processing system that implements these rules. This is with a broader objective of designing an oncology-based system to support cross laboratory, cross paradigm, and cross modal integration of brain functional data.

The next paper “Statistical modeling and analysis of laser-evoked potentials of electrocorticogram recordings from awake humans,” by Z. Chen et al., provides a comprehensive analysis of electrocorticogram recorded using invasive laser stimulation. Both averaging and single trial laser-evoked potentials (LEP) have been considered. Then the LEPs have been extracted from both types of trials, and the variations in power, amplitude, and latency have been studied using probabilistic modeling, factor analysis, independent component analysis, wavelet domain and quantitative and qualitative analyses.

The seventh paper “A Novel constrained topographic independent component analysis for separation of epileptic seizure signals,” by Min Jing and Saeid Sanei addresses a constrained source separation method which exploits the correlation among the nearby brain sources as well as characteristics of the seizure signals in space and frequency domains to highlight the sources of interest. In this method the space-frequency characteristics of the data is utilized as the constraint term in the update equation of the topographic ICA system. The results clearly show that the synchronously generated seizure sources are grouped together.

The next paper, “Clustering approach to long term spatio-temporal interactions in Epileptic Electroencephalograph,” by A. Hegde et al., attempts to identify the spatio-temporal interactions of an epileptic brain using an existing non-linear dependency measure based on a clustering approach. The mutual interactions have been analyzed using an index measure based on a self-organizing map (SOM) network. The results report a long term structural connectivity related to various seizure states. In addition, the authors have aimed at developing engineering tools to determine spatio-temporal groupings in a multivariate epileptic brain.

The ninth paper, “Automatic Seizure Detection based on Time-Frequency Analysis and Artificial Neural Networks,” by A. T. Tzallas et al., uses an artificial neural network system for detection of epileptic seizures from a set of features estimated from time-frequency domain EEG data.

Next paper, “Canonical decomposition of ictal scalp EEG and accurate source localisation: Principles and simulation study,” by M. De Vos et al., uses a dipole-based method for localization of epileptic seizure sources. In this method a canonical decomposition procedure extracts the seizure source by a three-way model assumption.

The eleventh paper, “The implicit function as squashing time Model, a novel parallel nonlinear EEG analysis technique distinguishing mild cognitive impairment, Alzheimer's disease and normal aged subjects with high degree of accuracy,” by M. Buscema et al., introduces an ANN-based method in which the MCI and AD can be classified based on the spatial information content of the restino EEGs. In this procedure the ANNs do not use EEGs as the input; rather, the inputs for the classification are the weights of the connections within the ANN to generate the recorded EEG data. The introduced TWIST system selects the best features.

The last paper, “The P300 as a marker of waning attention and error propensity,” by Avijit Kumar Datta, Rhodri Cusack, Kari Hawkins, Joost Heutink, Christopher Rorden, Ian Robertson, and Tom Manly, studies and examines the variation of P300 ERP with respect to the error in responding to the stimuli. During the course of this research it has been found that errors are associated with significant reduction in the amplitude of preceding P300, and the fluctuations in P300 amplitude across the task formed a reliable associate of individual error propensity, supporting its use as a marker of our sustained control over action.

